# Masked Hypertension in Healthy Children and Adolescents: Who Should Be Screened?

**DOI:** 10.1007/s11906-023-01260-6

**Published:** 2023-08-28

**Authors:** Tomáš Seeman, Terezie Šuláková, Stella Stabouli

**Affiliations:** 1 Department of Pediatrics, Charles University Prague, 2nd Medical Faculty, V Úvalu 84, 15006 Prague, Czech Republic; 2 Department of Pediatrics, University Hospital Ostrava, Ostrava, Czech Republic; 3 Department of Pediatrics, Medical Faculty, University of Ostrava, Ostrava, Czech Republic; 4 1st Department of Pediatrics, School of Medicine, Faculty of Health Sciences, Aristotle University Thessaloniki, Hippokratio Hospital, Thessaloniki, Greece

**Keywords:** Masked hypertension, MH, Blood pressure, BP, Ambulatory blood pressure monitoring, ABPM, Left ventricular hypertrophy, LVH

## Abstract

**Purpose of Review:**

The goal is to review masked hypertension (MH) as a relatively new phenomenon when patients have normal office BP but elevated out-of-office BP. Firstly, it was described in children in 2004. It has received increased attention in the past decade.

**Recent Findings:**

The prevalence of MH in different pediatric populations differs widely between 0 and 60% based on the population studied, definition of MH, or method of out-of-office BP measurement. The highest prevalence of MH has been demonstrated in children with chronic kidney disease (CKD), obesity, diabetes, and after heart transplantation. In healthy children but with risk factors for hypertension such as prematurity, overweight/obesity, diabetes, chronic kidney disease, or positive family history of hypertension, the prevalence of MH is 9%. In healthy children without risk factors for hypertension, the prevalence of MH is very low ranging 0–3%.

**Summary:**

In healthy children, only patients with the following clinical conditions should be screened for MH: high-normal/elevated office BP, positive family history of hypertension, and those referred for suspected hypertension who have normal office BP in the secondary/tertiary center.

## Introduction

### History of Masked Hypertension

The term masked hypertension (MH) has been firstly introduced by Thomas G. Pickering in 2002 [1]. Pickering proposed to use this term to describe an unusual phenomenon of elevated out-of-office but normal office blood pressure (BP). If the patients with MH are already treated by antihypertensive drugs, the term “masked uncontrolled hypertension” should be used to separate them from untreated patients with MH. The first study dealing with MH in children was published by the Japanese group of Matsuoka et al. in 2004 [2].

### Definition of Masked Hypertension

At the beginning, the researchers used the definition as normal office BP but elevated daytime ambulatory BP without taking into account the night-time BP values [1–4]. Later on, the definition changed to normal office BP but elevated daytime and/or night-time BP during 24-h ambulatory blood pressure monitoring (ABPM), thereby including also the patients with isolated nocturnal hypertension (INH) in the category of MH [5]. Therefore, MH may be diagnosed by ABPM on the basis of several scenarios, including:

- Isolated daytime hypertension (IDH)
- Isolated nocturnal hypertension (INH)
- Combined day- and night-time hypertension (DNH)

**Fig. 1:**
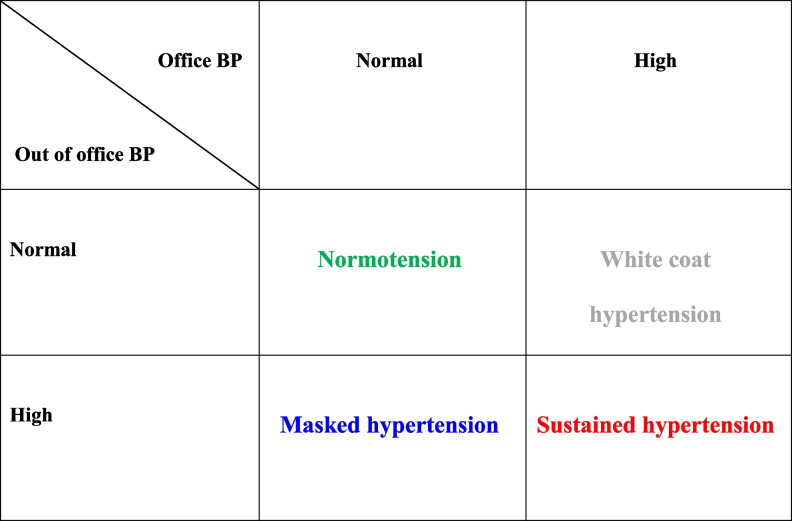
Blood pressure categories based on results of office and out of office blood pressure

When using both methods of BP measurement, namely, OBP and ABPM, we thus have four categories of BP level: normotension, hypertension, white-coat, and masked hypertension (Fig. 1).

### Methods of Out-Of-Office Blood Pressure Measurement for Diagnosing of Masked Hypertension

Either ABPM or home blood pressure monitoring (HBPM) can be used for diagnose MH [6–9]. In children and adolescents, there is reasonable agreement between ABPM and home BP measurement (HBPM). In a large study Stergiou et al. showed an agreement between these two out-of-office BP monitoring in 80% of cases, MH in 11% of children based on ABPM and in 8% based on HBPM [10]. However, this study as well as that of Wuhl et al. showed that HBPM has lower sensitivity and lower positive predictive value in comparison to ABPM for the diagnosis of MH [10, 11]. HBPM has further limitations, mainly the lack of night-time measurements to detect masked INH. Neither of the current European and American pediatric guidelines recommends HBPM for diagnosing of MH in contrary to ABPM [12, 13]. Therefore, ABPM seems to be the reference method of out-of-office BP measurement for the diagnostics of MH over HBPM in children [12–14].

Previously, MH defined by ABPM has been based on daytime BP only; today, there is a consensus of the ESH Working Group for ABPM to include also night-time and 24-h BP values for the definition of MH; however, the American guidelines do not mention 24-h BP values [13, 15].

### Prevalence of Masked Hypertension in Different Pediatric Populations

The prevalence of MH in different pediatric populations differs widely between 0 and 60% based on the population studied, definition of MH, or method of out-of-office BP measurement.

### Children with Chronic Kidney Diseases (CKD)

Masked hypertension is a prevalent phenomenon in children with CKD of all stages. In the large prospective observational CKiD study, MH has been detected in 38% of 366 children with CKD stages 2–4 [16]. Furthermore, it was associated with left ventricular hypertrophy, and the recent AAP Clinical Practice Guidelines recommend that children with CKD should be regularly monitored by ABPM and echocardiography [13].

Children with CKD due to various etiologies and even normal renal function (CKD stage 1) are also at risk for MH, but ABPM may be less likely to performed in children with CKD stage 1 than stages 2–5.

Atypical hemolytic uremic syndrome (aHUS) very often presents acutely also with hypertension. In the nationwide database in India, it has been demonstrated that also at the long-term children with aHUS often suffer from hypertension, 38% of them from masked hypertension [17].

Autosomal dominant polycystic kidney disease (ADPKD) is the most common hereditary kidney disease with high prevalence of ambulatory hypertension in which MH is present in 14% of pediatric patients [18].

In the autosomal recessive form of polycystic kidney disease (ARPKD), MH has been found in a most recent study in only 5% of patients, apparently because of the high prevalence of sustained hypertension evident also by office BP measurements (94%) [19].

Children with solitary kidney (estimated prevalence 1:3.000 children) are at increased risk of office hypertension especially those with signs of chronic kidney injury such as albuminuria/proteinuria or decreased GFR [20]. These spare data suggest that children with a solitary kidney and evidence of chronic kidney injury are at higher risk of masked hypertension (6–13%) [21, 22] than children with a healthy solitary kidney and no signs of chronic kidney injury [23]. However, these suggestions need more research with higher number of patients.

Renal scarring, often associated with vesicoureteric reflux (reflux nephropathy), is a common cause of pediatric hypertension and ambulatory, but not office blood pressure correlates with the grade of scarring [24]. Therefore, it is not surprising that MH is also prevalent in children with renal scarring ranging from 5 to 32% [24, 25].

The phenomenon of MH was also present in pediatric survivors of Wilms tumor [26]. Thirty four percent of them showed MH in a prospective cohort study. Interestingly, none of the ten children with MH had LVH. The authors speculated that this might be because of the opposing effects of anthracycline chemotherapy and arterial hypertension on left ventricular mass. Chemotherapy-related cardiotoxicity reduces left ventricular mass and causes heart failure while arterial hypertension increases left ventricular mass.

Limited data indicate that children with glomerulopathies are also at increased risk for MH. In children with IgA nephropathy, the most common glomerulopathy in adults and children, 11% had MH in one retrospective study [27]. Interestingly, ambulatory but not office BP was associated with the severity of histopathological findings on renal biopsy. Sarkar et al. found in children with frequent relapsing nephrotic syndrome, 16% having MH with longer duration of frequently relapsing disease, and higher BMI being correlated with ambulatory blood pressure [28]. Children with focal segmental glomerulosclerosis (FSGS) from the CKiD study demonstrated MH in 46% of cases [29]. Children with glomerulopathies are often not labeled as having CKD 2–4, and thus, we might speculate that MH is likely under-detected in these populations. From the above-mentioned studies, it is clear than even children with CKD stage 1 and thereby normal renal function are at risk of having MH.

Children with CKD stage 5 on chronic hemodialysis or peritoneal dialysis have also high prevalence of MH being present in 12–37% of children [30, 31]. Moreover, OBP was not representative of ambulatory BP underscoring the importance of ABPM also in dialyzed children.

In pediatric kidney transplant recipients, MH was first specifically documented by Paripovic et al. [32]. The authors demonstrated MH in 24% of transplanted children. Another study showed that nocturnal hypertension is the predominant form of post-transplant hypertension [33]. Recently, Hamdani et al. found MH in 29% of pediatric patients after kidney transplantation which was independently associated with left ventricular hypertrophy and worse allograft function emphasizing the deleterious effect of MH on the heart and kidney graft [34, 35]. In further BP studies, MH has been found in 1–38% of investigated children [36, 37]. Generally, the prevalence of MH in both dialyzed and transplanted children is higher than in any other previously investigated pediatric population. Therefore dialysis and kidney transplantation seem to be major risk factors for the presence of MH in children, and dialyzed and transplanted children should undergo regular ABPM.

### Children with Other Solid Organ Transplants or Hematopoietic Stem Cell Transplantation

Masked hypertension is highly prevalent also in children after liver transplantation (26–47%) suggesting the risk factors for MH are mainly transplantation-associated and not associated with a specific transplanted solid organ [38]. Similarly high prevalence of MH has been found also in pediatric patients after hematopoietic stem cell transplantation [39, 40].

In pediatric heart transplant recipients, masked hypertension has been found in 60% of the patients at 5 years after transplantation with the majority of cases having isolated nocturnal hypertension [41•]. However, in a prospective follow-up study, pediatric patients who underwent cardiac surgery for congenital heart defects did not demonstrate masked hypertension, despite the high prevalence of abnormalities by ABPM (48%) mainly non-dipping status (13%) [42]. The finding was consistent both in those with acute kidney injury (AKI) and non-AKI history in the post-surgery period.

### Children with Cardiovascular and Hematological Disorders

In pediatric patients with heart disease or chronic conditions/syndromes accompanied with cardiac or vascular abnormalities, a high prevalence of masked hypertension has been frequently reported [43–50].

Increasing literature in children after successful aortic coarctaction (CoA) repair shows that masked hypertension may be revealed in office normotensive patients with the reported prevalence reaching up to 47% [43]. The largest cohort study evaluated 110 children following successful CoA repair [50]. Masked hypertension was revealed by ABPM in 46% of the newly diagnosed hypertensive patients, while masked uncontrolled hypertension was found in 20% of the treated patients. These studies validate the recommendations for regular surveillance with ABPM in this population.

Sickle cell disease (SCD) is associated with increased risk of cardiovascular disease, although office BP levels have been reported to be normal. On the contrary, masked hypertension has been consistently reported in prospective cohorts of children with SCD that underwent ABPM with prevalence ranging from 6 to 35% among studies [44–47]. In a cohort of 52 children with SCD, masked hypertension was found in 35% of the patiens [46]. In children with sikcle cell disease, MH was not associated with microalbuminuria, while non-dipping was pointing out on an association of non-dipping phenomenon and the early marker of hypertensive nephropathy. Masked hypertension was present in two (6%) patients while the majority of patients (60%) had abnormal nocturnal dipping. In children with SCD, masked hypertension is most likely secondary to renal or vascular consequences of SCD. Indeed similar prevalence of masked hypertension (18%) and elevated cfPWV (carotid-femoral pulse wave velocity) was found in children with SCD (all having HbS/β-thalassemia) and high-risk controls (children referred for evaluation to hypertension clinic) [45]. All of the children with SCD and MH had nocturnal hypertension (three of them isolated nocturnal).

### Children with Diabetes

The prevalence of pediatric-onset diabetes is increasing rapidly worldwide. Poor glycemic control and prolonged diabetes duration in combination with numerous other traditional cardiometabolic risk factors including hypertension may lead to the early development of microvascular complications and cardiovascular disease (CVD). CVD is connected with increased prevalence of morbidity and mortality in young adulthood in both types of diabetes [48]. Pediatric populations with type 1 (T1D) and type 2 (T2D) diabetes are classically underdiagnosed and undertreated with hypertension being one of the main drivers for CVD.

#### Type 1 Diabetes

Recent reports showed that the prevalence of hypertension in T1D pediatric population based on office blood pressure (BP) measurement may be as high as 26–44%, depending on age, study population, or reference values/guidelines [49]. However, the prevalence of HTN in children with T1D based on ABPM is less clearly reported [66]. Several ABPM studies included only small groups of diabetic children (70–200, i.e., not population-based). The prevalence of ambulatory hypertension in these ABPM studies ranged 25–28% [50, 51]. Interestingly, nearly half of the children with ambulatory hypertension presented with masked hypertension (10–12%). Similarly to adults, true normotension in T1D children is not guaranteed by office blood pressure measurement only. Therefore, true (ambulatory) hypertension is underdiagnosed and undertreated, and ABPM is recommended in all diabetic children to assess out-of-office blood pressure and to reveal masked hypertension [50–52].

Masked hypertension in T1D patients is associated with longer duration of T1D, higher insulin requirement, and unstable glycemia [53••]. Isolated nocturnal hypertension, a subtype of MH, was a predictor of carotid intima media thickness, another sign of hypertension-mediated organ damage (HMOD) [54]. Lurbe et al. confirmed an association of the increase in nocturnal systolic BP and development of albuminuria during the follow-up [55]. Thus, nocturnal hypertension and MH in T1D youth are associated with early signs of hypertension-mediated organ damage.

Cardiac functional abnormalities in T1D children are associated with body mass index, disease duration, poor glycemic control, albuminuria, retinopathy, or increased BP [56]. Unfortunately, no study has evaluated the direct influence of MH on cardiac dysfunction in diabetic children.

#### Type 2 Diabetes

In adults with T2D, up to one-third of office normotensive patients have MH, which is associated with an increased albuminuria, left ventricle wall thickness, and impaired diastolic function [57]. Moreover, MH is also prevalent in offspring of patients with T2D (29%) and is associated with reduced coronary flow reserve and left ventricular diastolic dysfunction [58].

In children with T2D, the prevalence of office hypertension ranges 25–67% for pooled prevalence and cumulative incidence [59]. Unfortunately, the studies frequently reported only office BP without data on ambulatory BP. Therefore, blood pressure phenotypes such as masked hypertension of white-coat hypertension could not be determined in T2D children.

### Obese Children

In adults, the prevalence of MH seems to be higher in obese (31%) than in non-obese patients (6%) [60, 61]. Among African Americans, without antihypertensive treatment, metabolic syndrome was associated with MH [62].

The prevalence of obesity among children is steadily increasing worldwide, particularly in developed countries. The prevalence of MH in obese children and adolescents is reported between 9 and 16% and of isolated nocturnal hypertension up to 18% [63, 64]. Waist circumference and BMI are independent risk factors of pediatric MH [63, 64]. Larger waist circumference or higher level of obesity is associated with higher 24-h daytime and night-time BP and higher prevalence of office and ambulatory hypertension [63, 64]. Therefore, in children with severe obesity, ABPM is recommended to screen for masked hypertension.

### Children with Other Chronic Illnesses

Children with obstructive sleep apnea (OSA) demonstrate hypertension more often than healthy children [65]. Due to the disturbed sleep, they are prone to have also MH (23%), mainly due to isolated nocturnal hypertension. Furthermore, Kirk et al. showed a significant association between BP and the marker of OSAS, namely, the apnea hypopnea index (AHI) [65].

In 25 children and adolescents with lupus erythematosus and normal office BP, Mazo et al. found MH in 16% of patients paradoxically without any case of LVH [66].

Findings from the Victorian Infant Collaborative Study suggest that children born extremely preterm or extremely low birthweight (< 28 weeks, < 1000 g) might also be of increased risk of MH. These investigators demonstrated MH in 43% of children born extremely preterm or low birthweight at the median follow-up age of 18 years; however, in a control group of matched term healthy controls, the prevalence of MH was also high (36%) [67]. In one study from a developing country, children born very preterm and low birthweight (< 1500 g) had lower prevalence of MH (11%) compared to the former study; however, the absolute number of children with MH was very low (4 out of 46) [68]. All these findings have to be taken with great caution due to small number of children. More studies are needed to confirm this unexpected finding.

### Children with Risk Factors for Hypertension

In mixed cohorts of patients with different risk factors for MH, the prevalence of MH ranged from 8 to 11% [2, 69]. The prevalence of MH in children with several risk factors for hypertension has been studied recently [69]. Iturzaeta et al. found MH in 9% of children in a prospective study among more than 100 children with at least one risk factor for hypertension such as prematurity, low birthweight, history of umbilical cord cannulation, isolated transient high OBP in the past medical history, overweight/obesity, diabetes, chronic kidney disease, or positive family history of hypertension [69]. It is difficult to compare the prevalence between different studies due to different characteristics of the patients’ populations [2, 69].

### Masked Hypertension in Healthy Subjects

In adults, there are several studies on the prevalence of MH in general healthy population. The weighted mean prevalence from many studies using either ABPM or HBPM to define out-of-office BP is 11% [70••].

Contrary to adults, very few studies are available estimating the prevalence of MH in general pediatric population. The prevalence of MH in those studies ranged from 3 to 7%. In the Arsakeion School study, Stergiou et al. found MH based on HBPM in 3.3% of 765 healthy school subjects aged 6–18 years [71] with increased BMI and prehypertension/high-normal BP being independent predictors of MH. In another population-based study, Lurbe et al. demonstrated in nearly 600 children using ABPM, masked hypertension in 7.6% of them [3]. Obesity, parental history of hypertension, and higher ambulatory pulse rate were associated with MH. In a small study of 85 randomly selected school children, Kollios et al. found MH in 23% based on ABPM. Increased 24-h pulse wave velocity was present in MH subjects, as also noted in those with sustained hypertension [72].

The second type of studies from which we can estimate the prevalence of MH in healthy children is studies on different diseases with healthy controls. Recently, Dubey et al. and Yel et al. showed that MH was not present in any of 26 and 20 healthy controls [21, 44]. In another study, on obese children, Lurbe et al. could find MH only in 1.1% of healthy controls [63]. It is not known why findings in population-based studies differ from results found in research control groups.

Based on the low prevalence of MH in healthy controls, it appears that MH is an infrequent phenomenon in health children. Therefore, experts generally do not support screening healthy children with office normotension for MH. However, the presence of risk factor(s) for hypertension, such as chronic kidney disease, obesity, diabetes, or cardiovascular disorder, increases the risk of having MH [2, 4], and these children should be screened for the presence of MH by out-of-office BP measurement, preferably ABPM.

The prevalence of masked hypertension in different populations of children is summarized in Table 1.

**Table 1:** The prevalence of masked hypertension in different pediatric populations

Reference	Number of children	Population	Prevalence of masked hypertension	Method of out-of-office BPM	Comments
Stergiou et al. [71]	*n* = 765	Healthy children	3%	HBPM	Arsakeion School study
Lurbe et al. [63]	*n* = 180	Healthy controls	1.1%	ABPM	Study on obese children
Dubey et al. [44]	*n* = 26	Healthy controls	0%	ABPM	
Yel et al. [21•]	*n* = 20	Healthy controls	0%	ABPM	
Chaudhuri et al. [31]	*n* = 24	Dialysis	37%	ABPM	
Hamdani et al. [34•]	*n* = 77	Kidney transplants	29%	ABPM	
Bansal et al. [41•]	*n* = 34	Heart transplants	60%	ABPM	
Lurbe et al. [63]	*n* = 285	Obese and overweight	9%	ABPM	
Sulakova et al. [50]	*n* = 84	Type 1 diabetes	10%	ABPM	
Kollios et al. [72]	*n* = 85	Healthy children	23%	ABPM	School screening Kastoria study

*ABPM* ambulatory blood pressure monitoring, *HBPM* home blood pressure measurement

### Cardiovascular Consequences of Masked Hypertension

Masked hypertension in children and adolescents has been consistently associated with increased left ventricular mass index (LVMI) and left ventricular hypertrophy (LVH) [5, 16, 73, 74]. In a retrospective pooled analysis of data from two cross-sectional studies, including 163 adolescents recruited through hypertensive referral clinic or school screening, the risk for LVH was similar in children with masked and ambulatory (sustained) hypertension [75]. Similar results were reported in 85 children from a tertiary pediatric hypertension clinic. Children with masked hypertension presented higher LVMI than normotensive children and similar to those with ambulatory hypertension [5]. Seeman et al. found in their retrospective study that LVH was only present in children with INH, 58% of whom had masked hypertension [76]. Two pediatric studies reported data on persistence of masked hypertension during 4 years of follow up. Both highlight the presence of masked hypertension as significant prognostic factor for higher LVMI during follow-up compared to normotensive children [3, 77]. Lurbe et al. found that LVH at the end of follow-up period was only present in the persistent masked hypertension group or in patients with masked hypertension who progressed to sustained (true, ambulatory) hypertension (30% versus 0%; *P* = 0.01) compared to normotensive controls [3].

Several studies have assessed the association of masked hypertension with LVH in high-risk pediatric patients. In a cohort including patients with successful CoA repair, those with masked hypertension presented significantly increased pressure gradient in the aortic arch, increased LVMI, diastolic dysfunction with decreased E/A, reduced LV twist, and reduced basal septal longitudinal myocardial deformation properties compared to normotensives [78]. In children with CKD, poorly controlled hypertension in patients with non-dialysis CKD emerges as a strong determinant of LVH and was detected in one-third of the patients with sustained ambulatory hypertension and in 20% of children with masked uncontrolled hypertension [16]. In multivariable analysis, masked and confirmed hypertension present similar odds ratios and were the strongest independent predictors of LVH. Similarly, in a cohort of 221 children after kidney transplantation, multivariate analysis showed that abnormal ambulatory BP (masked or sustained hypertension combined) was an independent predictor for LVH among patients not receiving antihypertensive treatment but not in those with controlled hypertension [35].

In a cross-sectional study, including 85 children randomly selected from a school-based screening study, masked hypertension was associated with higher pulse wave velocity (PWV) over the 24-h period compared to children with WCH and normotension. Ambulatory hypertension and masked hypertension were independent predictors of 24-h PWV after adjustment for age, sex, and BMI *z*-score [72]. Stabouli et al., despite the association of masked hypertension with increased LVMI, failed to show positive association of masked hypertension with carotid intima media thickness (cIMT) [5]. However, in non-dialysis CKD patients from the 4C study cohort, those with isolated nocturnal hypertension (77% of them having masked hypertension) had increased risk for LVH, elevated cIMT, and PWV, but the presence of isolated nocturnal hypertension remained significant predictor only for elevated cIMT [79].

The detrimental neurological effects of ambulatory hypertension with emphasis on masked hypertension have been highlighted in limited studies. In a cohort of 42 children with SCD, masked hypertension was reported in 25% of them. All the masked hypertensive patients had nocturnal hypertension. The presence of masked hypertension was higher in children with SCD and either a silent cerebral infarct or stroke documented by MRI compared to those with no neurological complications (55% vs 12%) [47]. In cohort of 116 children with primary hypertension from a tertiary pediatric hypertension clinic, 21 (18%) presented masked hypertension with INH being the most prevalent MH phenotype (20/21) [80]. Children with nocturnal hypertension had lower performance in executive function tests with night-time systolic BP being an independent predictor of poor performance in several domains of executive function.

### Renal Consequences of Masked Hypertension

In adult patients, there are several studies showing that MH is associated not only with negative cardiac consequences but also with early hypertensive renal damage, specifically with albuminuria [81, 82].

In children with MH, there are virtually no data on albuminuria. Therefore, it needs to be elucidated whether MH is associated with albuminuria also in pediatric patients with primary hypertension as it has been already demonstrated in adult population.

### Predictors of Masked Hypertension

Masked hypertension may be caused by greater increase of ABP in comparison to OBP or unexpectedly low OBP. Several factors may have a greater impact on ABP than on OBP and thereby may cause MH. They can be divided into BP factors, clinical conditions/disorders, physiological/behavior factors, and demographic/lifestyle factors [14, 83]:

1. **BP level**

- OBP level that is just under the 95th percentile: In healthy children, prehypertension/high-normal BP (BP 90–95th percentile) was an independent predictor of MH [71]. Also in children with chronic kidney disease (CKD), high-normal OBP/prehypertension/elevated OBP predicted the presence of MH [84]. Similarly to children with CKD, office SBP at the 85th was the greatest predictor of MH also in children born with very low birthweight with a sensitivity and specificity around 80% [68]. These findings correspond nicely with the results of the PAMELA study where masked hypertensive adult patients had an average OBP of 129/84 mm Hg which was just below the cut-off for high-normal BP/prehypertension [85]. Moreover, in a study of children seen for preventive health care, Lurbe et al. found that most of those with MH had OBP values that were less than the high-normal range of 90–95th percentiles [3]. Patients with transiently high BP who have normal OBP at the secondary/tertiary center might be at increased risk of having MH [6, 10].
- ABP level that is just above the 95th percentile poses an increased risk of MH especially in persons with high daytime physical activities as they can have normal OBP [86]. These subjects with transiently elevated out-of-office BP due to high job/school stress can have normal OBP if OBP is measured under recommended rest conditions [6].

2. Clinical disorders that are associated with increased risk of MH such as increased body weight/obesity [3, 63, 71], OSAS [65], diabetes [2, 50], or chronic kidney disease [16].
3. Children with normal office BP but presence of hypertension-mediated organ damage (HMOD) [5].
4. Family/lifestyle predictors of MH such as parental history of hypertension [3] and male gender in children [87], high sodium intake in diabetics [88], high physical activity during the day, or sleep disturbances in adults [89].

Recently, Hung et al. invented a prediction model composed of six predictor variables such as office systolic and diastolic BP, mean arterial pressure, or pulse pressure using machine learning to predict masked and masked uncontrolled hypertension in adults [90]. Most recently, researchers from the CKiD study group suggested a machine learning-based prediction of masked hypertension among children with chronic kidney disease [91•]. The investigators indicated that performance of ABPM was not necessary in those with systolic OBP < 20th percentile and diastolic OBP < 80th percentile. Routine use of ABPM was suggested for children with OBP above these levels.

### Pathomechanisms of Masked Hypertension

The pathomechanism of MH is not yet fully elucidated. Risk factors associated with masked hypertension in adults are increased sodium intake, salt and fluid retention, or chronic kidney disease. Elevated BP during exercise is also present more frequently in transplanted patients with MH [92•].

The pathophysiological mechanisms of MH need to be understood at an individual level in order to develop effective tailored therapy [83].

### Reproducibility of Masked Hypertension

The most recent meta-analysis has demonstrated that the reproducibility of MH is only slight to fair with ABP results having better reproducibility than that of OBP; therefore, the poor reproducibility of MH may be the result of variable OBP measurements [93••]. However, this most recent large meta-analysis included adult studies only. Therefore, an ABPM/HBPM-based strategy should be established for diagnostics and therapy of children with MH. Furthermore, based on data largely from studies in adults, the prevalence of MH declines with repeated ABPMs suggesting that a correct diagnosis of MH should be based on at least two ABPMs and might be called “persistent masked hypertension” [14].

### Persistence of Masked Hypertension and Progression to Sustained Hypertension

The ABP phenotype including MH may change over time [3, 87, 94, 95]. Lurbe et al. showed in their longitudinal follow-up study that MH phenomenon persisted in nearly 18% of children. 49% of them became normotensive (ABP normalized) while in 31% of them masked hypertension progressed to sustained hypertension at a median follow up interval of 36 months (OBP increased into hypertensive levels). Interestingly boys progressed more often (50%) than girls (17%) [87]. Therefore, many experts suggest that MH may be a precursor of sustained hypertension [6, 87]. The sexual dimorphism noted in the propensity for progression to sustained hypertension may be related to the greater pubertal increase of daytime systolic BP in boys (3.5 mm Hg) in comparison to girls (0.7 mm Hg) and the overall higher prevalence of ambulatory hypertension in males than in females [3].

### Recommendations for Masked Hypertension Screening in Children

In adults, there is a general agreement that subjects who are at high risk of having masked hypertension should be screened [7, 14]. The American and European hypertension guidelines recommend the following adult populations to be screened for MH [8, 96]:

- Individuals with repeated OBP 120–129/75–79 mm Hg
- Individuals with OBP 130–139/85–89 (high-normal BP/prehypertension), with HMOD or at high cardiovascular disease risk

In children, the recent pediatric European and American hypertension guidelines recommend to screen for masked hypertension in children with CKD, with type 1 and 2 diabetes, obesity, after solid organ transplantations, with repaired coarctation of aorta starting 12 years after the surgery, with HMOD and normal office BP, and hypertensive response during treadmill or other exercise test [12, 13].

It is our practice to recommend that in addition to the children with the above disorders, healthy children with the following clinical conditions should be screened for MH:

- High-normal/elevated OBP (90–95th percentile) [13].
- Positive family history of hypertension [5].
- Children with history of elevated OBP but normal OBP upon evaluation at secondary/tertiary center [6].

Conversely, we recommend against masked hypertension screening in the following children:

- Healthy children without any risk factor for hypertension [21, 44, 63].
- Children with CKD and systolic OBP < 20th percentile [91•].

Recommendations on populations and criteria for screening of masked hypertension in healthy children are given in Table 2.

**Table 2:** Suggested indications of screening for masked hypertension in healthy children

**Healthy population to be screened**	**Healthy population not to be screened**
High-normal/elevated office BP [71]	Healthy children without any risk factor for hypertension [21, 44, 63]
Positive family history for hypertension [3, 63]	
Transiently elevated OBP [10, 69]	

*BP* blood pressure, *OBP* office blood pressure

### Management of Masked Hypertension

There are no published randomized controlled trials (RCT) on the treatment of MH in adults. However, there are currently two ongoing RCTs investigating the impact of treatment of MH, namely ANTI-MASK and MASTER study [13, 97]. The preliminary results of the ANTI-MASK trial have been presented as a congress abstract. The authors concluded that antihypertensive treatment was beneficial for patients with MH in terms of HMOD protection and regression.

The ACC/AHA guidelines recommend lifestyle modifications [96]. On the contrary, the recent ESC/ESH guidelines recommend also pharmacologic treatment in patients with MH and acknowledge that there is minimal evidence to support this recommendation [8].

In children, the most recent pediatric hypertension guidelines of the ESH and AAP give no treatment recommendations specifically for children with MH [12, 13].

Because ambulatory hypertension, either together with office hypertension (sustained hypertension) or with normal office BP (masked hypertension), is associated with increased cardiovascular and renal risk, we and others regard it as a clinical condition which warrants further investigation (blood tests, echocardiography, testing for albuminuria), follow-up, and intervention [4]. The question of which interventions should be started in a child with MH is yet to be answered; we think that lifestyle measures such as body weight reduction in overweight/obese children, age-appropriate sodium intake (e.g., < 2300 mg per day for adolescents), and appropriate physical exercise similarly to children with high-normal/prehypertensive OBP who are from the definition also non-hypertensive are reasonable interventions to target the lifestyle risk factors for hypertension and cardiovascular diseases. Similar recommendations are given also in the adult guidelines [8, 96].

There are no prospective randomized controlled trials in children with MH similar to children with office hypertension. Therefore, there is no evidence whether antihypertensive drug therapy may normalize the MH and improve cardiovascular risk profile in children with MH.

We recommend that antihypertensive drug therapy should be initiated in all children with MH if they have:

- Hypertension-mediated organ damage
- Secondary (renal) hypertension
- Diabetes
- Symptomatic hypertension.

in accordance to the recent pediatric guidelines for office hypertension [12, 13].

Lurbe has suggested that persistence of MH for 1 year in spite of lifestyle changes may be, as in children with office hypertension, an indication for BP-lowering treatment, especially in children and adolescents with a positive family history of hypertension [4, 98]. Awazu suggested salt restriction as a primary non-pharmacological intervention since high salt intake is suggested to be one of the pathomechanisms of MH [99].

## Areas for Further Research

Further studies are needed in the field of pediatric masked hypertension to confirm the prevalence and predisposing factors for MH in the general population as well as the risk factors for persistence and progression from MH to sustained hypertension and indications for screening. Furthermore, interventional randomized controlled trials on both lifestyle and pharmacological treatments of MH are also needed.

## Conclusions

Masked hypertension is a relatively new phenomenon that conveys an increased risk of HMOD and long-term progression to sustained hypertension. It is prevalent in children with risk factors for sustained hypertension such as obesity or CKD. Therefore, in children with these clinical conditions, out-of-office BP monitoring (ideally by ABPM) should be regularly performed even if office BP is normal to unmask potential masked hypertension. On the contrary, masked hypertension is rare in healthy children with normal office BP, and therefore, ABPM should not be performed in these children.
