# Correction to: Masked Hypertension in Healthy Children and Adolescents: Who Should Be Screened?

**DOI:** 10.1007/s11906-023-01271-3

**Published:** 2023-10-10

**Authors:** Tomáš Seeman, Terezie Šuláková, Stella Stabouli

**Affiliations:** 1 Department of Pediatrics, Charles University Prague, 2nd Medical Faculty, V Úvalu 84, 15006 Prague, Czech Republic; 2 Department of Pediatrics, University Hospital Ostrava, Ostrava, Czech Republic; 3 Department of Pediatrics, Medical Faculty, University of Ostrava, Ostrava, Czech Republic; 4 1st Department of Pediatrics, School of Medicine, Faculty of Health Sciences, Aristotle University Thessaloniki, Hippokratio Hospital, Thessaloniki, Greece

**Correction to: Current Hypertension Reports (2023) 25:231-242** 10.1007/s11906-023-01260-6

The article “Masked Hypertension in Healthy Children and Adolescents: Who Should Be Screened?”, was originally published electronically on the publisher’s internet portal on 28 August 2023 with error on the tagging of the author names (first names and last names were interchanged).

The correct tagging of the author names are as follows:

- Author 1
  - First Name: Tomáš
    Last Name: Seeman
- Author 2
  - First Name: Terezie
    Last Name: Šuláková
- Author 3
  - First Name: Stella
    Last Name: Stabouli

The original article has been corrected.

